# Widespread distribution and altered pain processing in head and neck cancer survivors at long-term after treatment

**DOI:** 10.1007/s00520-023-07846-9

**Published:** 2023-06-14

**Authors:** Sofía Hernández Hernández, Vanessa Gabriela Jerviz Guía, Javier Martín Núñez, Alejandro Heredia Ciuró, Alba Navas Otero, Esther Díaz Mohedo, Marie Carmen Valenza

**Affiliations:** 1 Department of Physiotherapy, Faculty of Health Sciences, University of Granada, Av. De La Ilustración, 60, 18016 Granada, Spain; 2 Department of Physiotherapy, Faculty of Health Sciences, Ampliación de Campus de Teatinos, University of Malaga, 29071 Málaga, Spain; 3 Oncological Radiotherapy Service of the Hospital PTS, Clínico San Cecilio University Hospital, 180061 Granada, Spain

**Keywords:** Head and neck, Cancer, Pain, Radiotherapy, Hypersensitivity

## Abstract

**Purpose:**

Radiotherapy (RT) treatment in head and neck cancer (HNC) patients may induce long-term sequels as pain, which nowadays is not fully understand. Therefore, there is a need of characterization of pain features in HNC to enhance after oncology treatment management. Head and neck cancer survivors develop chronic pain after radiotherapy treatment. The purpose of the current study is to evaluate the presence of pain, pain distribution, and pain processing by means of patient reported outcomes and quantitative sensory testing.

**Methods:**

Pain pressure threshold (PPT), temporal summation (TS), Brief Pain Inventory (BPI), Widespread Pain Index (WPI), The Disabilities of the Arm, Shoulder and Hand (DASH) questionnaire, and EuroQol5D5L were assessed in 20 head and neck cancer survivors (sHNC) and 20 health-related sex and age-matched controls.

**Results:**

The sHNC present lower PPT values in both the affected and non-affected side than did the healthy controls, especially in the widespread pain in the body, an altered TS in both affected and non-affected side and lower scores in quality of life and arm dysfunction.

**Conclusions:**

Following radiotherapy treatment after 1 year, sHNC present widespread pain, hypersensitivity in the radiated area, altered pain processing, upper limb affection, and a QoL diminution. These data provide evidence that a peripheral and central sensitization is happening in sHNC. Future efforts should focus on preventing pain after oncologic treatment. The comprehension about pain and its features in sHNC enhance health professional understanding and allows to tailor an optimal patient-targeted pain treatment.

## Introduction

Head and neck cancer (HNC) includes the malignant tumors mainly related to squamous cell carcinomas of the oral cavity, pharynx, and larynx being the seventh most common cancer worldwide in 2018 [1]. The mean characteristics related to diagnosis is being 50 years old, more frequent in men, alcohol and tobacco consumption, and presence of human papillomavirus [2]. The therapeutical proposal consisting in surgery, radiotherapy (RT), and/or chemotherapy has reached a high survival rate, ranging between 58 and 66%. Surgery and RT represents both the most curative treatment option, both in early and advanced stage of the disease [3].

Ionizing irradiation causes damage in normal tissues located in the field of radiation; in this line, HNC patients use to cope with radiation-related changes in the oral mucosa, salivary glands, taste, dentition, periodontium, bone, muscles, and joints effects. These adverse effects include oral mucositis, fatigue, facial disfiguration, and functional impairments related to eating, swallowing, and speaking [4]. Additionally, to those sequelae, some studies have reported frequent pain in HNC survivors, reaching a prevalence of 15 to 40% [5].

Those early and late sequelae of head and neck cancer patients have a large impact on the quality of life, but surprisingly, the pathogenesis of many of the oral sequelae of head and neck RT is not fully understood. Concretely, the innervation and the presence of various anatomical structures in a confined space can partially explain the values of pain in HNC. While some of the short-term sequelae naturally disappear, persistent pain can be present in 8 to 60% of head and neck cancer survivors (sHNC) [6].

Despite significant clinical relevance, chronic pain in sHNC seems to be under-considered and under-treated in long-term survivors, with some critical issues that some authors have pointed out [7]. First, there is no consensus about its features in terms of body distribution and pain processing. Second, pain studies in sHNC reported only pain intensity with no details of tissue damage and peripheral and central sensitization (CS). Finally, clinical related conditions have reported effects in pain-related cancer, but little is known about that in sHNC [6].

To clarify the pain profile in this setting through detailed description of how patients report the presence, distribution and processing of pain may provide indications that can have implications for clinical practice and research [8]. For this reason, the primary goal of the present study was to describe the features of persistent pain in sHNC. We further examined differences between sHNC with pain and healthy age- and sex-matched controls in terms of pain experience, pain distribution, pain pressure threshold (PPT), and temporal summation (TS).

## Materials and methods

### Samples

Eligible patients were ≥ 18 years of age, had a diagnosis of head and neck cancer, had completed RT and/or chemotherapy treatment 1 year prior to assessment, and were able to read and verbally communicate with the interviewer. Subjects reporting dementia or mental illness were excluded.

A control group was formed by healthy age- and sex-matched volunteers who responded to advertisements. They were excluded if they have suffered cancer or if they had any systemic disease.

The study was approved by the Biomedical Ethic Investigation Committee of Granada (Spain). Patients were recruited from the Radiotherapy Service of San Cecilio University Hospital (Granada, Spain), from November 2021 to June 2022, and conducted in accordance with the Declaration of Helsinki [9]. All patients were informed about the study procedure, and informed consent was obtained from all individual participants from whom identifying information is included in this article.

### Measures

#### Demographic and clinical measures

Patients completed a demographic questionnaire concerning sex, age, race, and severity of pain. Medical records were reviewed for disease and treatment information as tumor location, cancer stage at evaluation, coadjutant treatment, and medication consumption.

#### Pain characteristics and distribution

The Brief Pain Inventory (BPI) measures both pain intensity and pain interference. The values range from 0 to 10, being 0 “no pain” and 10 “imaginable worst pain.” It also assesses pain relief, pain quality, and patient perception of the cause of pain [10].

The Widespread Pain Index (WPI) measures body pain presence in 19 body regions over the last 7 days. One point is added for each body area where the patient refers to pain. Higher scores indicate higher pain dispersion [11].

#### Quantitative sensory testing outcomes

PPT was measured by means of a hand-held pressure algometer (Model Mark-10 M3-20 Series) which has a 1-cm^2^ rubber tip and a range from 0 to 12 kg. The tip was placed over a pre-defined point and continuous pressure of 1 kg/s was applied until the subject reported when the sensation of pressure changed into pain. This process was first applied at the forearm to verify that the subject had understood it [12, 13].

This testing was assessed bilaterally at eight points: trapezius, temporal, masseter, zygapophysial joint C5-C6, supraclavicular fossa, carpal tunnel, and anterior tibial. In healthy subjects, the affected side was taken as the irradiated homolateral side, while the contralateral side was taken as the healthy side. They were defined as proximal points within the diagnostic area of head and neck and distal points those located at distance. Three measurements were conducted, and the mean PPT was calculated [14–16].

TS was assessed in both left and right extensor digitorum. That pressure was applied repeatedly and continuously 15 times in the same location in the forearm, during 3 and 5 s. The patient’s pain was reported by asking how much pain the patient was experiencing from 0 to 100, being 0 “no pain” and 100 “worst pain at all” [17].

#### Health-related quality of life

The EuroQol5D5L is a questionnaire consisting of a system of five dimensions: mobility, self-care, usual activities, pain/discomfort and anxiety/depression, and a thermometer-like visual analog scale (VAS) anchored by 0 “worst imaginable health” and 100 “best imaginable health.” Responses to these items can be converted into a single measure of health utility using preference-based weights [18].

#### Upper-limb function

The Disabilities of the Arm, Shoulder and Hand (DASH) questionnaire uses a system of 30 items, where patients attribute scores from 1 to 5. Higher scores reflect higher disability [19].

### Statistical analyses

The data obtained from the evaluations were stored in an Excel database. They were analyzed using the Statistical Package for Social Sciences (SPSS) for Windows (version 26 IBM, Armonk, NY, USA). The normality of the data was assessed using the Kolmogorov-Semirnov test, while the homogeneity of variances was determined using Fisher’s test. For data with normal distribution, the Student’s *t*-test was used, and for nonparametric variables, the Mann-Whitney test was used.

Nominal values were expressed as frequencies and percentages. For continuous variables, they were expressed as mean and standard deviation. A value of *p* < .05 was used for significant differences.

## Results

### Demographic and clinical data

**Fig. 1:**
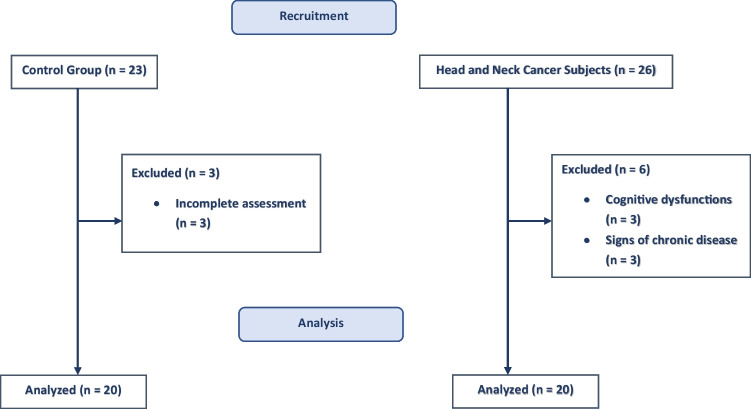
Patient recruitment flow chart

A total of 20 sHNC and 20 healthy-matched controls participated on the study. Figure 1 shows the patient recruitment flow.

The characteristics of the participants are summarized in Table 1. Of the 20 sHNC enrolled, 75% were male, and 25% were females. The study sample had an average age of 60 ± 13.92 years. The 70% of the sHNC present a squamous cell cancer, and the localizations of the tumor were 75% in larynx, 20% in pharynx, and 5% in the oral cavity.

**Table 1:** Characteristics of the participants

	sHNC (*n* = 20)	Healthy matched controls (*n* = 20)	*p* value
Age	59.95 ± 13.92	53.58 ± 15.48	.239
Gender			
Male	15 (75)	15 (75)	
Female	5 (25)	5 (25)	NA
Race			
Caucasian	20 (100)	20 (100)	NA
Histology			
Squamous	14 (70)	NA	NA
Location			
Oral cavity	1 (5)	NA	NA
Pharynx	4 (20)		
Larynx	15 (75)		
Staging			
1	4 (20)	NA	NA
2	7 (35)		
3	8 (40)		
4	1 (5)		
Coadjutant treatment			
Chemotherapy	8 (40)	NA	NA
Surgery	2 (10)		
No treatment	10 (50)		
Severity of pain			
Mild	5(25)	1 (5)	NA
Moderate	9 (45)	0 (0)	
Severe	6 (30)	0(0)	
Medication			
Opioids	14 (70)	NA	NA
Corticoids	7 (35)		
TCAs	0 (0)		
NSAIDs	9 (45)		
No medication	2 (10)		

The results of the EuroQol-5D, WPI, BPI, and DASH of both group of subjects are presented at Table 2. It shows the mean ± SD for the sHNC and for the control group.

**Table 2:** Comparison between groups in EuroQol5D5L, WPI, and BPI

	sHNC group (*n* = 20)	Control group (*n* = 20)	*p*
*EUROQL-5D-5L*			
Mobility	1.55 ± 1.05	1.08 ± 0.29	.15
Self-care	1.45 ± 0.99	1.00 ± 0	.13
Activities of daily living	1.45 ± 0.76	1.00 ± 0	.05*
Pain	2.10 ± 1.33	1.00 ± 0	.008*
Anxiety-depression	1.80 ± 1.01	1.00 ± 0	.01*
VAS (0-100)	72.84 ± 22.56	99.17 ± 2.89	< .001**
BPI			
BPI severity	4.37 ± 3.37	0.37 ± 0.56	.022*
BPI Interference	1.47 ± 2.12	0.38 ± 0.56	.091
BPI total	4.53 ± 4.20	0.84 ± 1.42	.041*
WPI	2.45 ± 3.43	0.42 ± 0.90	.05*
DASH	1.67 ± 0.97	1 ± 0	.028*

*VAS*, visual analog scale; *BPI*, Brief Pain Inventory; *WPI*, Widespread Pain Index; *DASH*, Disabilities of the Arm, Shoulder and Hand; data are expressed as mean ± SD; *p < .05; **p < .001

The EuroQol-5D5L showed significant differences between groups for the subscales: activities of daily living (*p* = .05), pain (*p* = .008), and anxiety-depression (*p* = .01). The VAS presented a significantly lower result (*p* < .001) in the sHNC group (72.84 ± 22.56) than the control group (99.16 ± 2.89).

For the BPI, sHNC group showed higher scores than the control group, presenting significant results for severity (*p* = .022) and the total score (*p* = .04). Twenty-five percent of the sHNC showed a lower pain of 4/10; meanwhile, only 5% of the healthy subjects presented a lower pain of 4/10.

In reference to the WPI, the sHNC group also showed significantly higher results (2.45 ± 3.43) than the control group (0.42 ± 0.90), with a significance of *p* = .05. Forty percent of the sHNC presented widespread pain; meanwhile, only 5% of the healthy subject showed widespread pain.

**Fig. 2:**
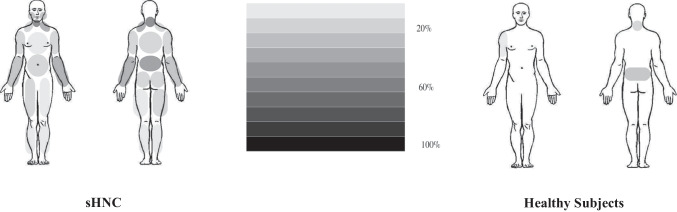
Pain areas referred by population

The regions with referred pain are represented in Fig. 2. The sHNC group referred to pain in neck, low back, and left upper limb. The healthy controls referred low back pain.

DASH scale shows a significant difference (*p* = 0.028) between groups, where higher values relate to higher interference with daily life.

Present pain areas in sHNC subjects and healthy subjects were percentage refers to amount of individuals who referred pain in the corresponding location.

The results of the PPTs are presented in Table 3 (mean ± SD). The sHNC group revealed lower values of PPT than the control group in all the evaluated points, presenting significant differences in both affected and unaffected site in the temporal, masseter, zygapophyseal joint C5-C6, and supraclavicular fossa thresholds. The trapezius of the affected side also presented significant differences (*p* = .04) between groups. No significant differences were seen between sHNC and the healthy subjects in both affected and unaffected sites in the anterior tibialis and carpal tunnel.

**Table 3:** Pressure-pain thresholds (kg/cm^2^) in HNC and healthy controls

		Control group (*n* = 12)	HNC group (*n* = 20)	*p*
Trapezius	Affected	5.01 ± 0.73	3.84 ± 1.78	.040*
	Unaffected	4.77 ± 1.02	4.13 ± 1.82	.201
Temporalis	Affected	3.90 ± 0.81	1.79 ± 0.83	< .001**
	Unaffected	3.44 ± 1.25	1.72 ± 0.65	< .001**
Masseter	Affected	2.87 ± 0.80	1.35 ± 0.49	< .001**
	Unaffected	2.96 ± 1.06	1.42 ± 0.70	< .001**
Zygapophyseal joint C5-C6	Affected	4.96 ± 1.18	2.23 ± 1.15	< .001**
	Unaffected	4.97 ± 1.46	2.27 ± 1.44	< .001**
Supraclavicular fossa	Affected	3.24 ± 0.94	1.79 ± 1.22	.001*
	Unaffected	3.26 ± 1.24	1.73 ± 1.04	< .001**
Anterior tibialis	Affected	5.38 ± 2.05	5.16 ± 2.29	.783
	Unaffected	5.70 ± 1.82	4.74 ± 2.16	.208
Carpal tunnel	Affected	4.97 ± 0.62	4.33 ± 2.31	.357
	Unaffected	4.63 ± 1.17	4.09 ± 2.38	.471

Data are expressed as mean ± SD; *p < .05; **p < .001

In the proximal area of the affected site, the lowest values of PPT in the sHNC were found in the masseter (1.35 ± 0.49), temporalis (1.79 ± 0.83), and supraclavicular fossa (1.79 ± 1.22). The highest PPT values were found in the anterior tibialis (5.16 ± 2.29) and in the carpal tunnel (4.33 ± 2.31). Same pattern was found in the unaffected site in the sHNC group.

**Fig. 3:**
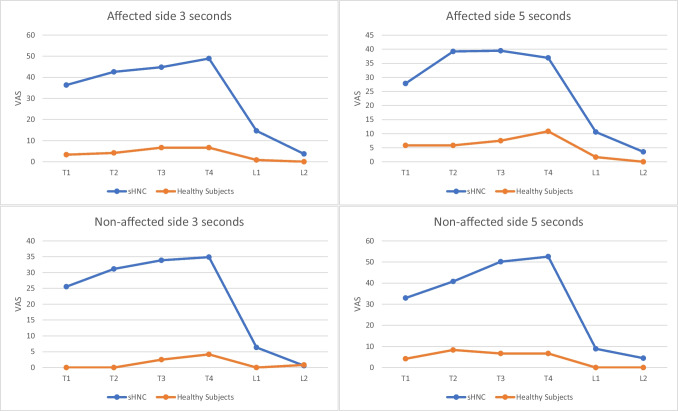
Temporal summation and latency in sHNC and healthy subjects

Figure 3 shows the differences between groups for latency and summation phenomenon. The PPTs for the forearm of the affected side of the sHNC group showed a mean of 2.73 ± 0.96 kg/cm^2^, and the forearm of the unaffected side showed a mean of 3.04 ± 1.60 kg/cm^2^. The control group showed a mean of 4.65 ± 1.28 kg/cm^2^ in the affected side and 4.75 ± 1.33 kg/cm^2^ in the unaffected side.

TS values were higher in the sHNC at any evaluation point compared to healthy subjects, being significative all of them (*p* ≤ .001). TS showed a slightly upward progression trend, with a rapid decrease in patient-reported pain values once the latency was assessed.

## Discussion

The main objective of the present study was to describe the features of persistent pain in sHNC. Additionally, we examined differences between sHNC with pain and healthy age- and sex-matched controls in terms of the pain experience, pain distribution, pain pressure threshold (PPT), and temporal summation (TS). In this line, we developed the pain assessment in sHNC using self-reported outcomes and quantitative sensory tests in order to submit as much information as possible about pain features in this population. Our results confirmed a higher presence of pain in sHNC patients, with a generalized distribution and accompanied by disturbed pain processing.

Overall, HNC patients continued to have pain 1 year after RT treatment, reaching a 75% of them presenting moderate-to-severe pain. Other studies developed on cancer survivors 1 year after diagnosis, reported more than 90% of patients with short-term pain symptoms related to their cancer or its treatment of whom more than 6% of adult cancer survivors reported pain intensity as “quite a bit/very much” 5 to 6 years post-diagnosis [20, 21]. Those results are different of ours, due the different cancer etiologies included. Other authors have yet reported that pain may be more common among certain subpopulations, such as breast, head and neck, and lung cancer survivors [22].

The most frequently reported areas of pain were neck, shoulder, forearm, and low-back, although generalized pain was also present. Our results are not consistent with the ones reported by Chua et al., where pain was only related to the tumor site area. This may be to their chosen sample, where more than 60% of sHNC had advanced tumor stage, compared to 5% of the sample in our study [23].

The results after the evaluation reveal that, compared with healthy controls, sHNC have lower PPT values in both affected and unaffected hemi-body in head, neck, shoulder, and arm. sHNC also respond to a wind-up phenomenon compared to healthy subjects and show a widespread distribution of pain, which is not limited to the radiated areas. The radiated area (temporalis, masseter, and supraclavicular fossa) was the most affected site in sHNC, while no significant differences were found in distal sites (tibialis anterior and carpal tunnel).

Compared to controls, widespread pressure hypersensitivity on both upper limb sides was detected in the sHNC with chronic pain. Other authors report similar findings in breast and colon cancer survivors [24, 25].

A randomized clinical trial carried out by Ortiz-Comino reported lower PPT values with significant differences among a healthy group in trigger points located in both proximal and distal sites (masseter, upper trapezius, supraclavicular fossa, and tibialis anterior). This difference may be due to their high percentage (90%) of subjects receiving combined treatment of RT and surgery for tumor removing, versus the 10% of the subjects receiving it in our study. Neck dissection is a comorbid condition which can lead to postoperative pain and the maintaining of peripheral sensitization [26]. The presence in sHNC of widespread pain hypersensitivity and altered pain processing mediated by the wind-up phenomenon support the view that there is a subjacent CS mechanism in sHNC reporting pain 1 year after radiation treatment.

When considering Qol 1 year after RT treatment, pain presence is still severe and can interfere in patients QoL. Additionally, other pain-related aspects in cancer patients have been proposed as relevant when exploring Qol like medication intake and psychoemotional distress [27, 28].

The values of the EuroQol5D5L reflect that the sHNC patients have poorer health-related quality of life. This can be due to the presence of pain, the high percentage of medication intake (about 90%), and the elevated scores in anxiety and depression. In a study conducted by Deschuymer et al., the authors found results in the same line than ours in breast cancer survivors who had received oncological treatment [29]. While the etiology of their sample was different, no other studies in sHNC patients evaluate Qol at long term after treatment.

We found arm disability significantly impaired in sHNC compared to healthy subjects. Several studies have shown upper limb functional affection in HNC patients, especially shoulder-related function [30]. It is believed that shoulder impairment results from damage of the accessory nerve after surgery of radiation. It can be associated with neck pain and other secondary effects in the shoulder, as adhesive capsulitis and myofascial pain in the trapezius, levator scapula, and rhomboid muscle [31].

TS was evaluated in both affected and non-affected side. sHNC referred that higher pain values were repetitive stimuli applied over the forearm. A significative difference related to TS differences between sHNC and healthy groups was found. Although assessing TS is a reliable way to evaluate altered pain processing, no studies in HNC patients were found to evaluate it. It has been proven to be a valid tool in other pathologies involving chronic pain as breast and endometrial cancer, hip arthroplasty, or fibromyalgia [32–34].

### Limitations

Since this was a cross-sectional design, it was not possible to establish cause-effect relationships and thus the contribution of treatment to the sensitization. In further studies, an evaluation before and after radiation treatment would be interesting to assess changes in pain over time. Moreover, the sHNC had different stages of disease, different location, and underwent different types of radiation treatments at the time of the evaluation. In the upcoming studies, pain may be studied in different groups of sHNC. Also, the present work presents a small sample of subjects, so studies with a larger number of sHNC should be carried out.

### Clinical implications

Radiation treatment may induce sensitization processes over sHNC, so further medical interventions must be made in order to reduce its effects. Pain in head, neck, and upper limb that appears after curative treatment could be treated. Multimodal interventions as physical therapy, exercise, and lifestyle changes help reducing the sensitization that occurs in sHNC. The knowledge of features of pain in this population could be of interest for applying the adequate dose and frequency of treatments by different health professionals.

## Conclusions

The present work reveals the existence of chronic pain, decrease PP values, an existing wind-up ratio process, and widespread pain in sHNC who underwent RT compared to healthy matched subjects. Hypersensitivity and hyperalgesia are present, suggesting, both peripheral and CS mechanisms in sHNC.

## Data Availability

The authors confirm that any datasets can be required for additional information.

## References

[1] Sung H, Ferlay J, Siegel RL (2021). Global Cancer Statistics 2020: GLOBOCAN estimates of incidence and mortality worldwide for 36 cancers in 185 countries. CA Cancer J Clin.

[2] Giraldi L, Leoncini E, Pastorino R (2017). Alcohol and cigarette consumption predict mortality in patients with head and neck cancer: a pooled analysis within the International Head and Neck Cancer Epidemiology (INHANCE) Consortium. Ann Oncol.

[3] Alterio D, Marvaso G, Ferrari A (2019). Modern radiotherapy for head and neck cancer. Semin Oncol.

[4] Zeng Q, Ling D, Chen W (2023). Family caregivers’ experiences of caring for patients with head and neck cancer: a systematic review and metasynthesis of qualitative studies. Cancer Nurs.

[5] Logan HL, Bartoshuk LM, Fillingim RB (2008). Metallic taste phantom predicts oral pain among 5-year survivors of head and neck cancer. Pain.

[6] Dugué J, Humbert M, Bendiane MK (2022). Head and neck cancer survivors’ pain in France: the VICAN study. J Cancer Surviv.

[7] Bossi P, Giusti R, Tarsitano A (2019). The point of pain in head and neck cancer. Crit Rev Oncol Hematol.

[8] Reich M, Leemans CR, Vermorken JB (2014). Best practices in the management of the psychooncologic aspects of head and neck cancer patients: recommendations from the European head and neck cancer society make sense campaign. Ann Oncol.

[9] Declaration of Helsinki World Medical Association Declaration of Helsinki Ethical Principles for Medical Research Involving Human Subjects 19886379

[10] Andersson V, Bergman S, Henoch I (2020). Benefits of using the Brief Pain Inventory in patients with cancer pain: an intervention study conducted in Swedish hospitals. Support Care Cancer.

[11] Dudeney J, Law EF, Meyyappan A (2019). Evaluating the psychometric properties of the Widespread Pain Index and the Symptom Severity Scale in youth with painful conditions. Can J Pain.

[12] Vanderweën L. Pressure algometry in manual therapy 10.1054/math.1996.0276 11440515

[13] Amiri M, Alavinia M, Singh M, Kumbhare D (2021). Pressure pain threshold in patients with chronic pain: a systematic review and meta-analysis. Am J Phys Med Rehabil.

[14] Schwartzman RJ, Grothusen JR (2008). Brachial plexus traction injury: quantification of sensory abnormalities. Pain Medicine.

[15] Calixtre LB, Oliveira AB, Alburquerque-Sendín F, Armijo-Olivo S (2020). What is the minimal important difference of pain intensity, mandibular function, and headache impact in patients with temporomandibular disorders? Clinical significance analysis of a randomized controlled trial. Musculoskelet Sci Pract.

[16] Rolke R, Baron R, Maier C (2006). Quantitative sensory testing in the German Research Network on Neuropathic Pain (DFNS): standardized protocol and reference values. Pain.

[17] Dams L, Haenen V, van der Gucht E (2022). Absolute and relative reliability of a comprehensive quantitative sensory testing protocol in women treated for breast cancer. Pain Med.

[18] Feng YS, Kohlmann T, Janssen MF, Buchholz I (2021). Psychometric properties of the EQ-5D-5L: a systematic review of the literature. Qual Life Res.

[19] Goldstein DP, Ringash J, Irish JC (2015). Assessment of the Disabilities of the Arm, Shoulder, and Hand (DASH) questionnaire for use in patients after neck dissection for head and neck cancer. Head Neck.

[20] Smith T, Stein KD, Mehta CC (2007). The rationale, design, and implementation of the American Cancer Society’s studies of cancer survivors. Cancer.

[21] Zucca AC, Boyes AW, Linden W, Girgis A (2012). All’s well that ends well? Quality of life and physical symptom clusters in long-term cancer survivors across cancer types. J Pain Symptom Manage.

[22] Mayer DK, Travers D, Wyss A (2011). Why do patients with cancer visit emergency departments? Results of a 2008 population study in North Carolina. J Clin Oncol.

[23] Chua KSG, Reddy SK, Lee MC, Patt RB (1999). Pain and loss of function in head and neck cancer survivors. J Pain Symptom Manage.

[24] Sánchez-Jiménez A, Cantarero-Villanueva I, Molina-Barea R (2014). Widespread pressure pain hypersensitivity and ultrasound imaging evaluation of abdominal area after colon cancer treatment. Pain Med (United States).

[25] Dams L, van der Gucht E, Meeus M (2021). Quantitative sensory testing in women after surgery for breast cancer: a systematic review and narrative synthesis. Clin J Pain.

[26] Terrell JE, Ronis DL, Fowler KE (2004). Clinical predictors of quality of life in patients with head and neck cancer. Arch Otolaryngol Head Neck Surg.

[27] Sharma Y, Mishra G, Parikh V (2019). Quality of life in head and neck cancer patients. Indian J Otolaryngol Head Neck Surg.

[28] McDowell L, Rischin D, Gough K, Henson C (2022). Health-related quality of life, psychosocial distress and unmet needs in older patients with head and neck cancer. Front Oncol.

[29] Deschuymer S, Nevens D, Duprez F (2021). Randomized clinical trial on reduction of radiotherapy dose to the elective neck in head and neck squamous cell carcinoma: results on the quality of life. Qual Life Res.

[30] Nilsen ML, Lyu L, Belsky MA (2020). Impact of neck disability on health-related quality of life among head and neck cancer survivors. Otolaryngol Head Neck Surg.

[31] McNeely ML, Parliament MB, Seikaly H (2008). Effect of exercise on upper extremity pain and dysfunction in head and neck cancer survivors: a randomized controlled trial. Cancer.

[32] Treister R, Honigman L, Berger A (2022). Temporal summation predicts de novo contralateral pain after cordotomy in patients with refractory cancer pain. Neurosurgery.

[33] Izumi M, Petersen KK, Laursen MB (2017). Facilitated temporal summation of pain correlates with clinical pain intensity after hip arthroplasty. Pain.

[34] Staud R, Weyl EE, Riley JL, Fillingim RB (2014) Slow temporal summation of pain for assessment of central pain sensitivity and clinical pain of fibromyalgia patients. PLoS One 9. 10.1371/JOURNAL.PONE.0089086 10.1371/journal.pone.0089086 PMC3928405 24558475

